# Testicular microlithiasis: Is there an agreed protocol?

**DOI:** 10.4103/0970-1591.33442

**Published:** 2007

**Authors:** R. Shanmugasundaram, J. Chandra Singh, Nitin S. Kekre

**Affiliations:** Department of Urology, Christian Medical College, Vellore - 632 004, Tamil Nadu, India

**Keywords:** Calcifications, microliths, tumor

## Abstract

This review addresses the issues on etiopathogenesis of testicular microlithiasis (TM), associated clinical entities, evaluation and follow-up of patients with TM. A literature search of Medline/PubMed was carried out using the keywords ‘testicular microlithiasis’ and ‘testicular calcifications’ for published data in English language on TM from 1970 to 2006. TM is an uncommon entity among adult males, resulting from intratubular calcifications. The reported incidence of TM is highly variable. With the increasing frequency of ultrasound examination in scrotal and testicular conditions and with the advent of high frequency transducers, TM is increasingly being reported. TM is associated with many benign and malignant conditions of testes but the possible association of TM with testicular cancer has been a matter of concern. Though a few sporadic cases of testicular malignancies have been reported, it is believed that a conservative approach is warranted in the absence of high risk factors, in view of the low risks for invasive cancers. There is no uniform protocol for the evaluation and follow-up of the patients with TM. Those with high risk factors like contralateral testicular tumour, chromosomal anomalies, gonadal dysgenesis, cryptorchidism and definite ultrasound pattern of TM should be advised to have further evaluation. Incidentally detected asymptomatic TM during ultrasound examination does not warrant aggressive measures and it can be followed with self examination.

Testicular microlithiasis (TM) is an entity of unknown etiology that results in formation of intratubular calcifications. It is often detected incidentally when scrotal ultrasonogram is done for various indications. Testicular microlithiasis are often multiple, uniform, small, echogenic polytopic intratubular calcifications without acoustic shadows. Despite the reports of association of TM and testicular tumor, there is no uniform consensus regarding the need for evaluation and method of follow-up of these patients. This review is focused on issues regarding the etiopathogenesis, clinical conditions associated with TM, methods of follow-up and about who should be followed? A literature search of Medline/PubMed was carried out using the keywords ‘testicular microlithiasis and ‘testicular calcifications’ for published data on TM from 1970 to 2006. Relevant literature was also obtained from various published updates and case reports.

## HISTORICAL BACKGROUND

Oiye described intratesticular calcifications in autopsy specimens as early as 1928.[1] In 1929, Blumensaat described similar intratubular bodies in postmortem specimens.[2] It was believed that the calcifications were degenerated spermatogonia in the lumen of seminiferous tubules. Azzopardi and Mostofi reported amorphous hematoxylin staining calcified bodies in dilated seminiferous tubules of patients with widespread choriocarcinoma.[3] These calcifications were found in close association with malignant cells. In 1970 Priebe and Garret first reported diffuse microcalcifications in the testis on a plain X-ray film in a four-year-old boy.[4] Prior to the development of ultrasound, diagnosis of testicular microlithiasis was done mainly by histology. Doherty *et al*. described ultrasonic appearance of testicular microlithiasis in 1987 as “innumerable tiny bright echoes diffusely and uniformly scattered throughout in the substance of testes” using high frequency transducer (10 MHz).[5] From there on, the interest in this entity increased.

## INCIDENCE

The true incidence of TM is unknown due to limited number of cases, significant differences in studied patient populations, diagnostic methods and definitions used and also due to undefined prevalence of TM in normal populations. Autopsy studies revealed testicular microliths in 0.04–11.8% of prepubertal boys and in 3% of adult males.[1,2,6] Sohval found calcified intratubular bodies in four of 59 (6.7%) testicular specimens of adults and children.[7] Nistal *et al.* reported TM in one of 618 (0.6%) testicular biopsy specimens performed in children with cryptorchid testes.[6] In a retrospective analysis of 1710 testicular sonograms of adults performed for various conditions, bilateral TM was demonstrated in 11 cases (0.6%).[8] In a prospective series involving ultrasound screening study for 1504 men between 18 to 35 years (mean 22.8 years) from the US army officer corps, Peterson *et al.* found the prevalence of testicular microlithiasis to be 5.6%.[9] African Americans were found to have a higher prevalence of 14% as opposed to whites with prevalence of 4%. However, the incidence of testicular tumors is higher in whites than in African Americans. Analysis of the geographical distribution of these cases showed a negative correlation of testicular microlithiasis with the incidence of testicular tumor in this study. In their follow-up report after more than four years presented at the American Urological Association (AUA) meeting in 2004 at San Francisco. USA,[10] they have not had a single case of testicular tumor in their study subjects with testicular microlithiasis. In another prospective study involving healthy male volunteers (17–42 years of age) using screening scrotal ultrasound scan, a prevalence of 2.4% (53 of 2179 ultrasound scans were identified to have TM) was noted by Serter *et al.*[11]

## ETIOLOGY

The origin of testicular microlithiasis is unknown. Numerous theories have been proposed including liquefaction of protoplasmic dendritus of a spermatocyte or coalescence of colloid droplets, ectopic oocytes in dysgenetic testes, displaced spermatogonia, undifferentiated or desquamated calcified cells, deposition of glycoprotein around the nidus of cell material sloughed into the tubular lumen and abnormal sertoli cells activity.[1–3,12–15] Staged development of microliths resembling crystal-matrix formation of urinary calculi was also suggested.[16] Vacuolized degenerating cells not phagocytized by sertoli cells were suggested to form the nidus of microlith within the tubular lumen. Vegni-Talluri *et al.* have described two zones within the microliths.[16] The central calcified zone is surrounded by a zone of concentrically layered collagen fibers. Further, the diminished capability of sertoli cells to phagocytize the degenerating cells was accounted to the proximity of carcinoma *in situ* (CIS) in testis.[17] Halley propounded the breakage of tubular basal membrane, possibly due to an immunological mechanism, to be the cause of TM.[18] Glycoproteins released from the basal membranes form the matrix and precipitation around the nidus forming microliths. Deranged chemical composition of certain mucosubstances is another propounded cause.[19] Extratubular origin from eosinophilic bodies in the tunica propria of seminiferous tubules was suggested by Nistal *et al.*[20] They described the presence of testicular microliths in pediatric patients with bilateral cryptorchidism.[20] The study of such calcifications supported the hypothesis that the mineralization process occurs according to the following stages: 1) accumulation of cellular debris in the tubular lumen, 2) deposition of concentric rings of glycoprotein surrounding the central core and 3) calcification of the glycoprotein lamellar material.

As the microliths are seen in testis as well as in extratesticular sites like the lungs and central nervous system, genetic alterations were thought to play a role in their development. Mutation in the SLC34A2 gene (Chr 4p15) is found to be seen in patients with pulmonary alveolar microliths. Male patients with this mutation are found to have testicular microliths as well.[21] But the definite etiology of testicular microlithiasis is yet to be found.

## CLINICAL PRESENTATION

Testicular microlithiasis is most commonly diagnosed as an incidental finding on high frequency (7.5 to 10MHz) testicular ultrasound.[19] It is seen in males of different age groups, from childhood to old age. However, it is rare in prepubertal boys and in older men more than 60 years of age. Usually, it is bilateral in distribution. Unilateral cases have been occasionally reported.[22] Mostly, the presentation is asymptomatic and is often diagnosed with imaging. There are reports of painful testicular microlithiasis.[23] The mechanism of pain was suggested to be distension of seminiferous tubules. Other conditions in which TM is diagnosed often are infertility and testicular tumors.

Testicular microlithiasis was identified in various conditions, which are listed in Table 1.

**Table 1:** Testicular microlithiasis with associated conditions[44]

Infertility Testicular atrophy Cryptorchidism Varicocele Hydrocele Torsion of testis and its appendages Epididymal cysts Male pseudohermaphroditism Hypogonadism Calcification of sympathetic nervous system and Brain Pseudoxanthoma elasticum Fragile X syndrome Down's syndrome Kleinfelter's syndrome Carney's syndrome (skin pigmentation and cardiocutaneous myxoma) Pulmonary alveolar microlithiasis Cystic fibrosis Non-Hodgkin's lymphoma Neurofibromatosis Multiple lentigines AIDS Asymptomatic

## DIAGNOSIS

### Histopathology

Microliths (also called as calcospherites) are spherical, elongated or ovoid in shape and are eosinophilic. Under the light and electron microscopy, microliths are found to consist of two zones: a central calcified zone and a multilayered envelope stratified collagen fibers. It is further covered with a thin fibrous capsule of spermatogenic epithelium.[16,24] It was suggested that calcium is present in oxalate, carbonate or inorganic covalently bonded states. The microliths usually give positive reaction for van Kossa stain indicating presence of calcium, but also stain strongly with Schiff periodic acid which is resistant to diastase digestion. Microliths may occupy 30 to 40% of the seminiferous tubules and range in size from 50 to 400 μM^2^;.[21] The Leydig cells are not typically affected by testicular microlithiasis[24] and the majority of the uninvolved seminiferous tubules often has abnormal spermatogonia and reduced luminal diameters.[6,12,16] Spermatogenesis may be halted at the first order spermatocyte while some patients presented with normal spermatogenesis.[12,23]

Renshaw classified the testicular calcifications apart from true ossifications seen in teratoma as two types: 1) Hematoxylin bodies 2) Lamellated calcifications. Hematoxylin bodies were specific for germ cell tumors. Lamellated calcifications were more common in germ cell tumors, but they also occurred in normal testes. Renshaw suggested that the pathological criteria for TM should include laminated calcifications.[25]

### Imaging

#### Ultrasound

**Figure 1:**
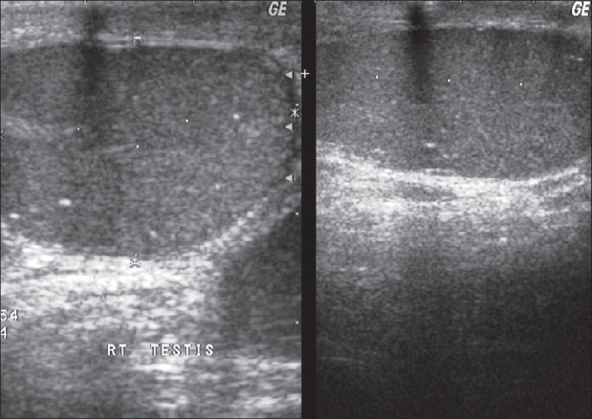
Testicular microlithiasis

Doherty described the classic sonographic findings of TM as “innumerable tiny bright echoes diffusely and uniformly scattered in testes” [Figure 1]. The ultrasound appearance is described as “snow storm” or “heaven full of stars” appearance.[5] Later reports confirmed that the microliths are confined to the testes alone and the epididymis and scrotum appeared normal.[6] Backus *et al.* demonstrated side to side variation in the number of echogenic foci in the primary peripheral distribution of calcifications in 12 of 42 patients (42%).[22] Highly echogenic foci were seen sonographically at the periphery of the testicular mass in 66% of germ cell tumors but no correlation was found between the pattern of calcification and the presence of neoplasm.[22] Hogarth *et al.* helped formalize the sonographic definition of TM that is still used today.[8] Ultrasound diagnosis is based on following criteria: 1) Greater than five calcifications per image field, 2) Calcifications less than 2 mm in diameter 3) Diffuse in nature 4) No acoustic shadowing and 5) No loss of testicular shape or volume. Testicular microlithiasis has been divided into classic TM (with five or more microliths on any single view) and limited TM (less than five microliths). It has been graded as minimal/mild (Grade I: 5 to 10 microliths), moderate (Grade II: 10 to 20 microliths) and severe (Grade III: >20 microliths) depending on the microliths count as seen in any single view.[26] There was a general trend of greater incidence of tumors with greater number of microliths, but there was no significant difference between the two subsets for either limited TM or classic TM.[27] Biopsy of the testes for detection of CIS based on the microliths count is controversial.

When limited TM is seen in the ultrasound, it should be differentiated from various conditions causing calcifications in testes as follows:[44]

1. Inflammatory: Orchitis, tuberculosis, sarcoidosis, focal scars and post-inflammatory granulomas.
2. Vascular: Arteritis, chronic infarction
3. Postoperative: Post-orchiopexy, sperm granulomas
4. Neoplastic: “Burned out” tumors, calcified sertoli cell tumors, following radiotherapy and chemotherapy.
5. Miscellaneous: Adrenal rests

Lenz *et al.* have devised a scoring system to describe the texture of testes based on ultrasound appearance in 444 asymptomatic men.[28] It is denoted in Table 2.

**Table 2:** Scoring system to describe testicular texture by Lenz *et al*.[28]

Score	Pattern of microcalcification		Prevalence % among population
1	Regular	}	68
2	Slightly irregular		
3	Moderately irregular		25
4	Very irregular, including echoes or microcalcifications		4
5	Tumor suspicion		0.7

The significance of this ultrasound scoring was assessed in the non-affected testis of 78 men with unilateral testicular cancer. The predictive value of Score 4 for the testes to contain CIS was 22.2% and the predictive value of a score different from 4 that the testes would not have CIS was 97.6%. Biopsy of the testes was recommended when a score of 4 or more was seen in testicular sonography.[29]

The sonographic findings of TM did not always correlate with histolopathological findings. In a series by Backus *et al*., calcification was present on pathological examination only in 10 of 22 patients who had TM on ultrasound.[22]

## CLINICAL ASSOCIATION

### Infertility

The relationship between TM and infertility is unclear. Since 30–40% of seminiferous tubules are obstructed with intratesticular concretions in patients with TM, obstruction of seminiferous tubules formed by sloughing degenerative tubular epithelium has been suggested as an underlying cause of TM in this condition.[6] The reported frequency of TM in patients with infertility or undescended testis ranges from 7–39%.[6,16] In studies by Janzen *et al.*[19] and Miller *et al.*,[30] 37 and 39% of cases of TM were associated with an undescended testis or sub fertility/infertility respectively. In another study with 159 infertile patients, microcalcifications were found in 10 (6.2%).[31] In this report the lesions were unilateral in all patients. Hobarth *et al.* reported oligo or azoospermia in 19% of the patients (3 of 16 patients) with TM respectively.[32] Fertility potential may be decreased by mechanical obstruction of seminiferous tubules with microliths, atrophy of uninvolved tubules with spermatogenic arrest or a combination of both.[4,12] It was suggested that microliths and infertility may have a common unidentified etiologic factor. Limited cases of testicular biopsy in patients with infertility and TM revealed microliths in 30–40% of the seminiferous tubules with obstruction of the tubular lumen, increased cytoplasmic swelling, vacuolization and atrophy of seminiferous epithelium.[24] Uninvolved segments present with a wide range of changes from low mean tubular diameter with hypospermatogenesis, maturation arrest at level of secondary spermatocyte stage, normal number of spermatogonia or even normal spermatogenesis.[23] With increased use of testicular sonography in the evaluation of infertile men, TM may be detected more often. The outcome of infertility has not been found to be different if TM is present. Hence incidental discovery of TM should not change the treatment of infertility.

### Testicular microlithiasis and malignancy

**Figure 2:**
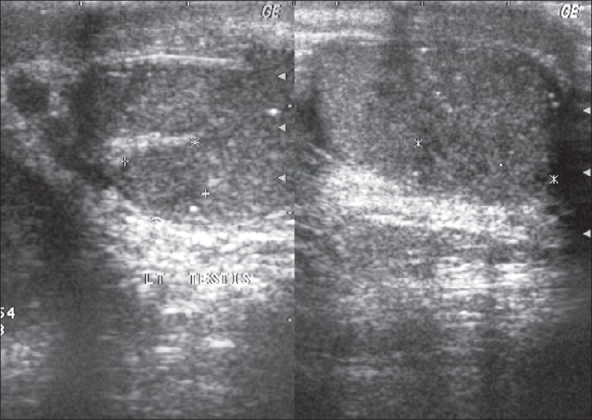
Seminoma associated with testicular microlithiasis

The evaluation of testicular biopsy and orchidectomy specimens has identified the coexistence of intratesticular microcalcifications and malignancy. Ikinger *et al.* found microcalcifications in 32 of 43 testicular tumors (74%) using mammographic technique. Microcalcifications were multiple, diffuse and grouped in 60% of the specimens and solitary calcified spots were seen in the remaining specimens. On the other hand, only eight of 49 (16%) specimens of benign testicular disorders revealed microcalcifications.[33] Janzen described a series of 11 patients with TM, two of whom had coexistent seminoma (18%).[19] Various other authors have shown same coexistence of testicular tumor in 17 of 42 patients (40%), five of 11 patients (45%), six of 16 patients (38%) and 29 of 63 patients (46%) with TM in their series respectively.[8,22,32,34] Derogee *et al.* diagnosed testicular tumor in only one patient of 31 patients during mean follow-up of 61.8 months. The same patient had bilateral undescended testes with previous treatment for testicular cancer signifying the importance of evaluation in the high-risk group.[34] Testicular tumors have been seen in four of 47(8.5%) patients with classic TM and not seen in patients with limited TM.[26] Figure 2 denotes the association of seminoma with microlithiasis.

The association between TM and testicular CIS has been well documented in a few series. In a retrospective study of testicular specimens with CIS but without tumors Kang *et al.* found TM in 14 of 36 (39%) specimens compared to 2.1% in a control group (*P*<0.001).[17] Songh *et al.* reported ipsilateral microlithiasis in 14 of 21(67%) patients with testicular CIS and considered focal clumped TM without testicular mass as an indicator for tumor.[35]

On the other hand, the results of a few prospective series on asymptomatic men with TM failed to prove development of interval malignancy. Janzen found no malignancy in six patients with classic bilateral TM.[19] Bennett performed follow-up sonography with a range of 0.5 to 5.6 years in 21 patients with classic and limited TM. None of his patients developed interval malignancy.[26] A few case reports support development of tumor in those who had established diagnosis of TM. Lawrentschuk has reported a case of bilateral TM with normal serum tumor markers developing classic seminoma at 12 months follow-up.[36] McEniff *et al.* reported a case of yolk sac tumor of testis developing in a 17-year-old boy with TM, four years after diagnosis of TM.[37]

Brazao *et al.* reported a 40-fold higher prevalence of CIS in men with bilateral TM compared to those without TM (20% vs. 0.5%).[38] Testicular microlithiasis has been linked to a common developmental defect of the seminiferous tubules and defined as part of the “Testicular dysgenesis syndrome” which comprises testicular cancer, genital abnormalities, sub-fertility and reduced sperm quality.[39] What so ever, the treatment of CIS is controversial. Treatment options for CIS include observation, radiation therapy, chemotherapy and orchidectomy. Persistence of CIS and occurrence of second germ cell tumors have been described after cisplatin-based chemotherapy.[40] Radiotherapy often makes these men infertile. It is believed that a conservative approach is warranted in view of the low risks of invasive cancer. High-risk patients require close observation, biopsy and CIS therapy to be given only after patients have understood all the risks and benefits involved.[41]

## FOLLOW -UP OF TESTICULAR MICROLITHIASIS

Since TM is occasionally associated with germ cell tumors, clinical and sonographic follow-up is recommended.[19,42] Serial physical examination, regular annual high resolution testicular sonography and chest X-ray are recommended to rule out malignancy. Serum tumor markers[43] and chromosomal analysis[30] were also recommended. Shenykin suggested routine testicular tumor marker determination as well as yearly scrotal ultrasound for patients with TM.[44] Duchek suggested chromosomal analysis in patients detected to have TM.[23] Miller recommended CT scan of the abdomen and chest to evaluate for extratesticular germ cell tumor.[30] Though biopsy was also recommended for all patients diagnosed to have TM,[30,45] the results of the limited studies and case reports available at the moment do not justify routine testicular biopsy in all patients with typical appearance of TM. There is no consensus on the necessity, interval and duration of follow-up and the diagnostic modality to be used.[9] The value of early diagnosis of CIS is still not confirmed and it has controversies due to protracted natural history, prolonged follow-up and unknown probability of developing invasive carcinoma. Relatively lesser impact on hormonal function and fertility following small risk of an invasive procedure like radical orchidectomy in case of detection of macroscopic tumor may not warrant an aggressive protocol like biopsy of all testes diagnosed to have TM. Moreover, the diagnosis of TM and leaving the patients who are at the prime of their productive (reproductive) period with anxiety of potential cancer and burden of prolonged follow-up of countless years need to be thought of. Testicular tumor is a treatable malignancy and it has a high cure even with nodal metastases. Only 5–10% present with extensive disease. With the use of better chemotherapeutic agents and radiotherapy, CIS can also be treated effectively but at the cost of decreased fertility.[39] The benefit of strict follow-up in patients incidentally diagnosed to have TM has not been documented. Peterson *et al.* showed that serum tumor markers in their asymptomatic population were normal.[9] Obtaining AFP, β-HCG and lactate dehydrogenase as a baseline, cost more than $16 per patient. Annual scrotal ultrasound examination in their facility cost $100. The economic burden of evaluating and following men with TM, who are in the 18–35 years age group, for the time that they are at risk for testicular cancer was estimated to be greater than $18 billion.[9] Reasons for recommending early detection of tumor in those who are diagnosed to have TM include possible presentation with disseminated disease, decreased quality of life and fertility after treatment for testicular tumors. But so far the evidence for routine biopsy in all patients is not convincing. Biopsy is possibly indicated in patients with clinical and radiological high-risk factors as denoted in Table 3.

**Table 3:** Indications for testicular biopsy in patients with Testicular microlithiasis

Ipsilateral tumor and TM in the contralateral testis[22,45]
TM with gonadal dysgenesis and other chromosomal anomalies[24]
TM in infertile men and patients with cryptorchidism or atrophic testis[17,45]
Focal, clumped and unilateral TM without mass[34]
Grade IV or V pattern in testicular ultrasonography[29]
Classic TM - Grade 3 in microlithiasis count (>20)[26]

TM - Testicular microlithiasis

If testicular malignancy is absent in the first evaluation, TM can be followed up with regular self-examination of testes. Further evaluation and biopsy is indicated only for the high-risk group. If any change is noted by the patient, serum markers may be performed. In case of elevation of tumor markers, biopsy can be done to rule out CIS provided there is intent to treat. In case of tumor detection in the first instance of follow-up, further management can be planned accordingly. Patients and their family members need to be educated about the possible association of testicular malignancy with TM, regular self-examination and prolonged follow-up.

## CONCLUSION

TM is a benign lesion seen uncommonly in the asymptomatic population of men between 20 and 50 years of age, with prevalence varying from 0.6–5.6%. The association with testicular cancer is a cause of concern for this uncommon lesion of unclear etiology. Careful evaluation and follow-up is advised in those at high risk of developing testicular cancer like cryptorchidism, infertility, testicular atrophy and contralateral testicular cancer. Biopsy should be done only if detection of CIS will be followed with treatment. Treatment of CIS itself has swung from chemotherapy, radiotherapy and orchidectomy to active surveillance. For patients with TM, who are asymptomatic and are not at high risk of development of CIS and invasive tumor, regular self-examination and prompt reporting to the physician in case of appearance of any new lesions should suffice. In the present scenario, TM detected during routine ultrasound evaluation for various scrotal conditions other than those with high risk does not warrant biopsy. The anxiety and economic burden that are imposed on patients with TM when prolonged follow-up is advised should be considered against the backdrop of a malignancy with excellent outcome.
